# Development and Validation of a Machine Learning–Based Early Warning Model for Lichenoid Vulvar Disease: Prediction Model Development Study

**DOI:** 10.2196/55734

**Published:** 2024-11-22

**Authors:** Jian Meng, Xiaoyu Niu, Can Luo, Yueyue Chen, Qiao Li, Dongmei Wei

**Affiliations:** 1 Department of Obstetrics and Gynecology West China Second Hospital Sichuan University Chengdu China

**Keywords:** female, lichenoid vulvar disease, risk factors, evidence-based medicine, early warning model

## Abstract

**Background:**

Given the complexity and diversity of lichenoid vulvar disease (LVD) risk factors, it is crucial to actively explore these factors and construct personalized warning models using relevant clinical variables to assess disease risk in patients. Yet, to date, there has been insufficient research, both nationwide and internationally, on risk factors and warning models for LVD. In light of these gaps, this study represents the first systematic exploration of the risk factors associated with LVD.

**Objective:**

The risk factors of LVD in women were explored and a medically evidence-based warning model was constructed to provide an early alert tool for the high-risk target population. The model can be applied in the clinic to identify high-risk patients and evaluate its accuracy and practicality in predicting LVD in women. Simultaneously, it can also enhance the diagnostic and treatment proficiency of medical personnel in primary community health service centers, which is of great significance in reducing overall health care spending and disease burden.

**Methods:**

A total of 2990 patients who attended West China Second Hospital of Sichuan University from January 2013 to December 2017 were selected as the study candidates and were divided into 1218 cases in the normal vulvovagina group (group 0) and 1772 cases in the lichenoid vulvar disease group (group 1) according to the results of the case examination. We investigated and collected routine examination data from patients for intergroup comparisons, included factors with significant differences in multifactorial analysis, and constructed logistic regression, random forests, gradient boosting machine (GBM), adaboost, eXtreme Gradient Boosting, and Categorical Boosting analysis models. The predictive efficacy of these six models was evaluated using receiver operating characteristic curve and area under the curve.

**Results:**

Univariate analysis revealed that vaginitis, urinary incontinence, humidity of the long-term residential environment, spicy dietary habits, regular intake of coffee or caffeinated beverages, daily sleep duration, diabetes mellitus, smoking history, presence of autoimmune diseases, menopausal status, and hypertension were all significant risk factors affecting female LVD. Furthermore, the area under the receiver operating characteristic curve, accuracy, sensitivity, and *F*_1_-score of the GBM warning model were notably higher than the other 5 predictive analysis models. The GBM analysis model indicated that menopausal status had the strongest impact on female LVD, showing a positive correlation, followed by the presence of autoimmune diseases, which also displayed a positive dependency.

**Conclusions:**

In accordance with evidence-based medicine, the construction of a predictive warning model for female LVD can be used to identify high-risk populations at an early stage, aiding in the formulation of effective preventive measures, which is of paramount importance for reducing the incidence of LVD in women.

## Introduction

In different time periods, lichenoid vulvar disease (LVD) has been variously named such as white spot disease, or vulvar white lesions. The term LVD was coined by the International Society for the Study of Vulvovaginal Disease in 1975. LVD is classified into 3 pathological types, namely vulvar squamous cell hyperplasia type, lichen sclerosus type, and mixed type [1,2]. It primarily manifests as mucocutaneous hypopigmentation of vulva to gray-brown or whitening, roughening of skin with lichenification or reduction of elasticity with atrophic thinning and chapping, and introital stenosis. It is characterized by pruritus or pain, regardless of seasons or day-night variations, with exacerbation often occurring at night [3]. The lesion locations include the labia majora and minora, the clitoral region, perianal area, and the pubic hairline. LVD is often recurrent, and in the later stage, it can lead to vaginal stenosis, causing dyspareunia and even carrying the risk of carcinoma, which severely impacts the physical and psychological well-being of the patients [4-6]. It is evident that Chinese women are often reluctant to seek medical care from formal health care facilities due to economic, cultural and traditional concept, and other factors. Clinical practice, both domestically and internationally, has shown that early warning, timely diagnosis, prompt intervention, and immediate treatment are effective strategies in preventing the development of LVD [6,7]. The early identification of high-risk individuals and proactive prevention of LVD have garnered increasing attention. However, current research on the early identification of risk factors for LVD remains inadequate, lacking systematic and comprehensive approaches. For instance, Sideri et al [8] conducted a case-control study with 75 cases and 225 age-matched controls to assess risk factors for LVD. The study collected data on personal characteristics and habits, gynecologic and obstetric history, general sexual habits, and selected dietary habits. The findings indicated a higher risk of vulvar lichen sclerosis (VLS) in parous women compared with nulliparous women, with no significant increase in risk correlating with the number of births. Smith and Haefner [9] highlighted the association of VLS with autoimmune conditions, genetic factors, hormone levels, and infections. These studies were limited to identifying high-risk factors and did not develop early warning models.

Given the complexity and diversity of LVD risk factors, it is crucial to actively explore these factors and construct personalized warning models using relevant clinical variables to assess disease risk in patients. Yet, to date, there has been insufficient research by scholars, both domestically and internationally, on the risk factors and warning models for LVD. In light of these gaps, this study represents the first systematic exploration of the risk factors associated with LVD. Using an evidence-based medicine approach, a warning model has been constructed, offering a tool for the early identification of high-risk individuals. Furthermore, this model has the potential to enhance the diagnostic and treatment capabilities of medical personnel in primary community health service centers. This holds great significance in mitigating overall health care expenditures and the disease burden. More importantly, our research is the first to apply machine learning techniques to predict LVD, filling a novel research gap in the field.

## Methods

### Study Candidates

The study incorporated 2634 female patients who attended the West China Second Hospital of Sichuan University from January 2013 to December 2017. Based on the results of their medical examinations, these patients were divided into 2 groups, that is, the normal vulvovagina group (group 0) consisting of 1080 cases and the LVD group (group 1) comprising 1554 cases. Inclusion criteria for the study encompassed female patients who were definitively diagnosed with LVD, were fully informed, voluntarily participated in the study, and could undergo long-term follow-ups. Exclusion criteria were as follows: (1) patients with mental illness history, cerebral infarction, or other conditions that may lead to communication difficulties; (2) patients in the acute phase of the disease; and (3) patients diagnosed with malignant tumors.

### Diagnosis of LVD

Per the 2021 European Guideline for the Management of Vulval Conditions, patients can be diagnosed with LVD according to the characteristic clinical manifestations and signs, including symptoms such as vulvar pain and itching (with severe cases leading to scratching), a burning sensation in the vulvovaginal area, abnormal vaginal discharge, dyspareunia, or even dysuria, along with signs such as vulvar skin keratinization hypertrophy, coarsening and thickening of the cortex or with part atrophy thinning, reduced or absent skin elasticity, and in severe cases, chapping and festering, hypopigmentation, or whitening of the skin [10]. In this study, however, the diagnostic criteria for LVD involve not only the characteristic clinical appearances but also require confirmation through biopsy. Conducting a biopsy before treatment is advantageous for establishing a definitive histopathological record for the diagnosis.

### Collection of Clinical Information

Based on previous domestic and international research findings combined with clinical experience, we selected and collected clinical information related to female LVD [11-14]. This included factors such as age, educational attainment, vaginitis, urinary incontinence, humidity of the long-term residential environment, type of long-term residence (urban or rural), spicy dietary habits, regular intake of coffee or caffeinated beverages, good sleep habits, daily sleep duration (<8 hours or ≥8 hours), diabetes mellitus, smoking history (≥100 cigarettes smoked in lifetime) [15], alcohol consumption, presence of autoimmune diseases, human papilloma virus status, presence of allergic diseases, menopausal status, hypertension, frequent seafood consumption, and income level.

### Statistical Analysis

**Figure 1:**
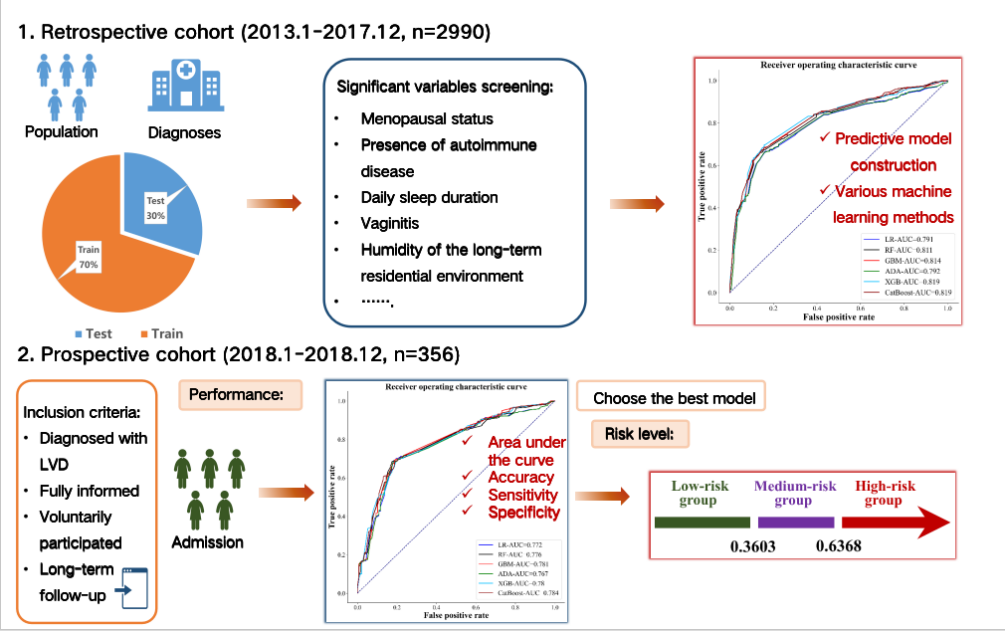
Study design. The early warning system model was built on the retrospective cohort (n=2990) and validated on the prospective cohort (n=356). LVD: Lichenoid Vulvar Disease; LR: Logistic Regression; RF: Random Forest; GBM: Gradient Boosting Machine; ADA: AdaBoost; XGB: Extreme Gradient Boosting; CatBoost: Categorical Boosting; AUC: Area Under the Curve.

In this study, the K-Nearest Neighbor imputer method was applied to fill in missing variables. To ensure the accuracy of the results after filling, variables with a missing rate greater than 50% (such as occupation, income level, educational attainment, age, and frequent consumption of seafood or high-fat foods) were excluded from modeling. Risk factors associated with female LVD were obtained after univariate analysis of all variables on the postfilled data. Variables that exhibited significance in the univariate statistical test were included in the recursive feature elimination for multivariate selection, and the area under the curve (AUC) value was highest when 10 variables were included [16]. Subsequently, these selected variables were used for machine learning modeling, resulting in the construction of logistic regression (LR), random forests (RF), gradient boosting machine (GBM), adaptive boosting (ADA), eXtreme gradient boosting (XGB), and categorical boosting (CatBoost) analysis models. The rationale for selecting these models lies in their exemplification of fundamental and archetypal algorithmic types within machine learning, LR for classification model, RF for bagging model, ADA for boosting model, and GBM for gradient boosting tree model. Widely applied across diverse domains and tasks, these algorithms consistently demonstrate robust performance. Furthermore, XGB and CatBoost represent advanced models that refine and enhance these classic algorithms, showcasing notable prowess, especially within the gradient boosting tree category (Multimedia Appendix 1) [17]. The predictive efficacy of these 6 models was assessed using receiver operating characteristic (ROC) curves, accuracy, sensitivity, and specificity. Based on the above best model, the correlation ranking between each variable and the target variables was investigated using SHapley Additive exPlanations (SHAP) plots. The details are shown in the study design workflow (Figure 1).

### Ethical Considerations

Ethical approval was granted by the Ethics Committee of West China Second Hospital of Sichuan University (2023179) on September 21, 2023. Informed consent was obtained from all participants. All procedures were performed in accordance with relevant guidelines.

## Results

### Univariate Analysis of Risk Factors for LVD

According to the results of case examinations, the study cohort was stratified into 2 groups, the normal vulvovagina group (group 0) with 1080 cases and the LVD group (group 1) with 1554 cases. The univariate analysis was performed, revealing statistically significant differences between the 2 groups in various factors, including vaginitis (*P*<.001), humidity of the long-term residential environment (*P*=.01), spicy dietary habits (*P*=.00), regular intake of coffee or caffeinated beverages (*P*=.00), daily sleep duration (*P*<.001), diabetes mellitus (*P*<.001), smoking history (*P*<.001), presence of autoimmune diseases (*P*<.001), menopausal status (*P*<.001), and hypertension (*P*<.001). Conversely, no statistically significant discrepancies were observed between the 2 groups in other baseline data, such as urinary incontinence (*P*=.07), type of long-term residence (*P*=.11), good sleep habits (*P*=.24), alcohol consumption (*P*=.42), and presence of allergic diseases (*P*=.52). Detailed findings are presented in Table 1.

**Table 1:** Univariate analysis of risk factors for lichenoid vulvar disease.

Parameter	Total (N=2634), n (%)	Group 0 (n=1080), n (%)	Group 1 (n=1554), n (%)	Chi-square	*P* value
Vaginitis	860 (32.65)	396 (36.667)	464 (29.858)	13.1	<.001
Urinary incontinence	275 (10.44)	98 (9.074)	177 (11.39)	3.4	.07
Humidity of the long-term residential environment	282 (10.706)	136 (12.593)	146 (9.395)	6.4	<.01
Type of long-term residence	2356 (89.446)	953 (88.241)	1403 (90.283)	2.6	.11
Spicy dietary habits	1365 (51.822)	601 (55.648)	764 (49.163)	10.4	<.001
Regular intake of coffee or caffeinated beverages	235 (8.922)	119 (11.019)	116 (7.465)	9.4	<.001
Good sleep habits	1607 (61.01)	644 (59.63)	963 (61.969)	1.3	.24
Daily sleep duration	921 (34.966)	263 (24.352)	658 (42.342)	89.9	<.001
Diabetes mellitus	112 (4.252)	15 (1.389)	97 (6.242)	35.7	<.001
Smoking history	86 (3.265)	55 (5.093)	31 (1.995)	18.4	<.001
Alcohol consumption	300 (11.39)	130 (12.037)	170 (10.94)	0.6	.42
Presence of autoimmune diseases	2070 (78.588)	689 (63.796)	1381 (88.867)	236.5	<.001
Presence of allergic diseases	229 (8.694)	99 (9.167)	130 (8.366)	0.4	.52
Menopausal status	896 (34.017)	141 (13.056)	755 (48.584)	356.7	<.001
Hypertension	50 (1.898)	31 (2.87)	19 (1.223)	8.4	<.001

### A Risk Model to Predict LVD

The establishment of the warning model begins with the foundation of massive data provided by large-sample retrospective clinical profiles, as the basis for evaluating numerous variables and screening out truly meaningful risk factors. Subsequently, the construction of machine learning models serves as the cornerstone for developing the early warning model. Further validation of the model’s accuracy is performed in clinical follow-up cohorts and prospective studies to establish the genuinely significant warning model. Since 2013, the pelvic floor group in West China Second Hospital of Sichuan University, has set up a big data cloud platform encompassing data from tens of thousands of patients with LVD, which provides a substantially big data foundation for the development of early warning and diagnostic model for LVD.

**Figure 2:**
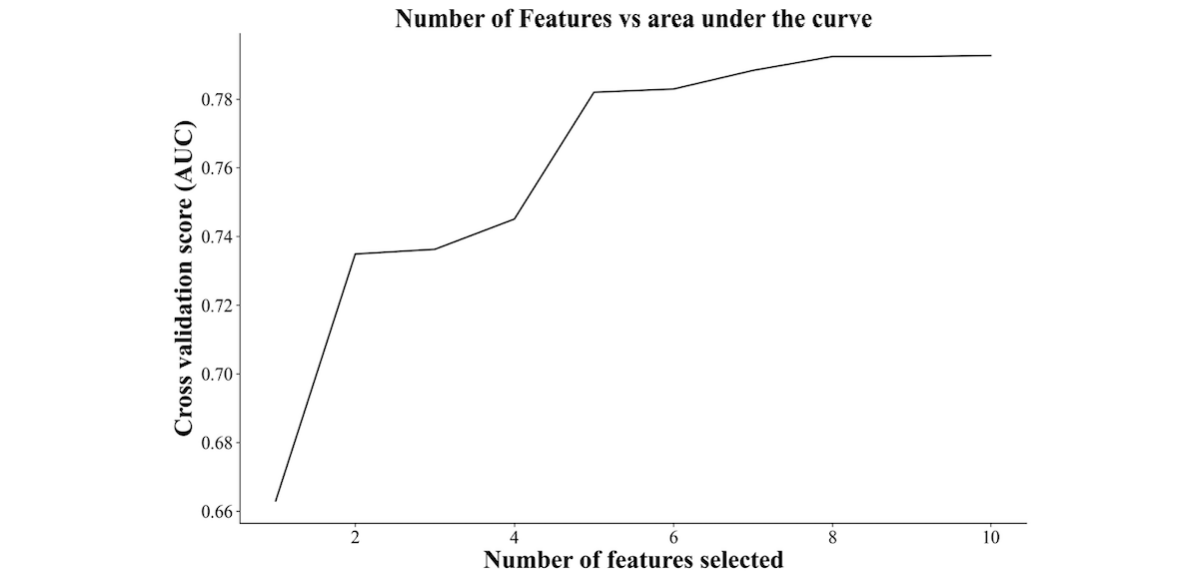
Recursive feature elimination variables screening curve for lichenoid vulvar disease (defined as group 1). AUC: Area under the curve.

**Figure 3:**
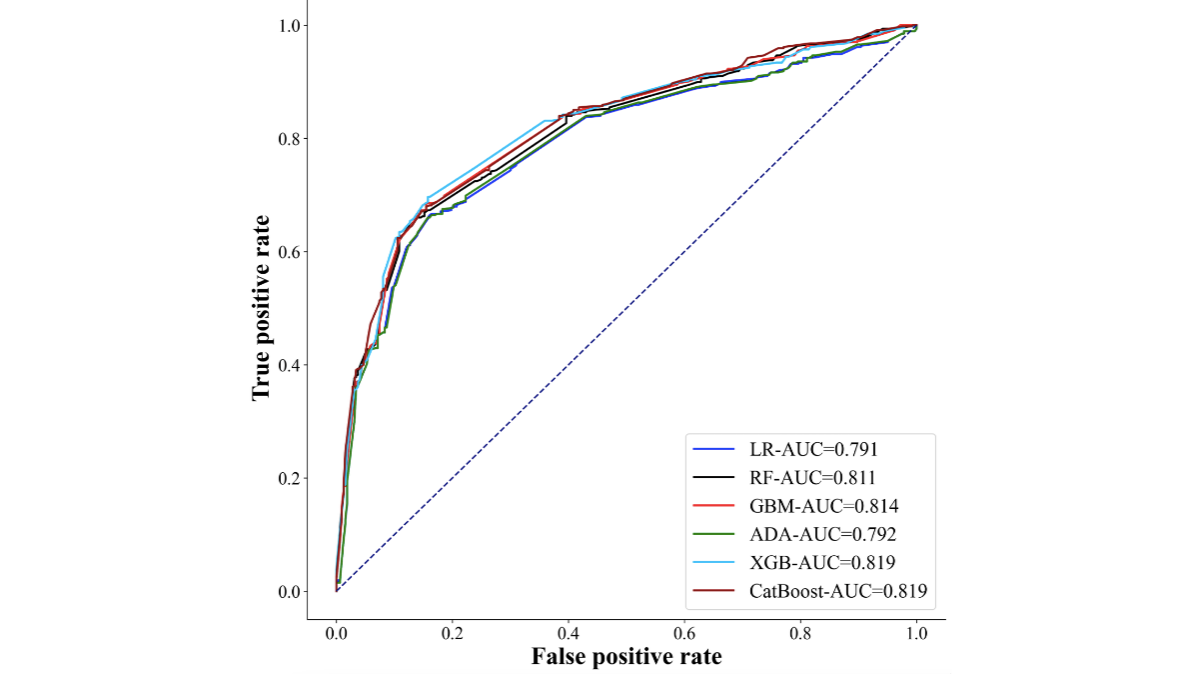
Receiver operating characteristic curves for 6 lichenoid vulvar disease prediction models. LR: Logistic Regression; RF: Random Forest; GBM: Gradient Boosting Machine; ADA: AdaBoost; XGB: Extreme Gradient Boosting; CatBoost: Categorical Boosting; AUC: Area Under the Curve.

The variables that showed significance in the univariate statistical tests were included in the recursive feature elimination multifactorial screening. As depicted in Figure 2, AUC was used as the evaluation metric, and the highest AUC score with 0.794 was achieved when 10 variables were included. In Table 2, the predictive models were constructed using various machine learning methods, including LR, RF, GBM, ADA, XGB, and CatBoost analysis models, based on the above selected 10 variables. Optimal parameters for each model were determined using grid search method combined with 5-fold cross validation. A comprehensive elucidation of the model principles and parameter selections is provided in the appendix. Results indicated superior performance of the GBM model, yielding an accuracy value of 0.747, sensitivity of 0.703, and specificity of 0.818. The ROC curves demonstrated that the AUC score of the GBM model was 0.814, as illustrated in Figure 3. In summary, the GBM warning model exhibited superior predictive efficacy, with higher values for the area under the ROC curve, accuracy, sensitivity, and specificity compared with the other 5 warning models.

**Table 2:** Results of the prediction efficacy assessment of 6 lichenoid vulvar disease prediction models.

Model^a^	Area under the curve, mean (95% CI)	Accuracy, mean (95% CI)	Sensitivity, mean (95% CI)	Specificity, mean (95% CI)	*F*_1_-score, mean (95% CI)
LR^b^	0.79 (0.765-0.818)	0.733 (0.703-0.752)	0.667 (0.629-0.703)	0.814 (0.777-0.86)	0.748 (0.717-0.768)
RF^c^	0.811 (0.784-0.839)	0.738 (0.712-0.767)	0.741 (0.703-0.773)	0.738 (0.691-0.78)	0.771 (0.735-0.796)
GBM^d^	0.814 (0.786-0.842)	0.747 (0.711-0.772)	0.703 (0.642-0.732)	0.818 (0.779-0.857)	0.766 (0.726-0.79)
ADA^e^	0.793 (0.763-0.825)	0.728 (0.705-0.764)	0.695 (0.661-0.733)	0.776 (0.736-0.815)	0.753 (0.721-0.785)
XGB^f^	0.818 (0.788-0.845)	0.751 (0.712-0.776)	0.672 (0.628-0.71)	0.862 (0.829-0.895)	0.761 (0.729-0.793)
CatBoost^g^	0.82 (0.791-0.847)	0.75 (0.722-0.778)	0.678 (0.648-0.713)	0.846 (0.818-0.882)	0.760 (0.73-0.787)

^a^The warning model was constructed using prediction models such as LR, RF, GBM, ADA, XGB, and CatBoost. The dataset was divided into a training set and a test set in a 7:3 ratio. The models were trained using the training set and evaluated using the test set. The numbers in parentheses represent the 95% CIs derived from 100 bootstrap iterations.

^b^LR: logistic regression.

^c^RF: random forests.

^d^GBM: gradient boosting machine.

^e^ADA: adaptive boosting.

^f^XGB: eXtreme gradient boosting.

^g^CatBoost: categorical boosting.

### Shapley Additive Explanations Correlation Analysis

**Figure 4:**
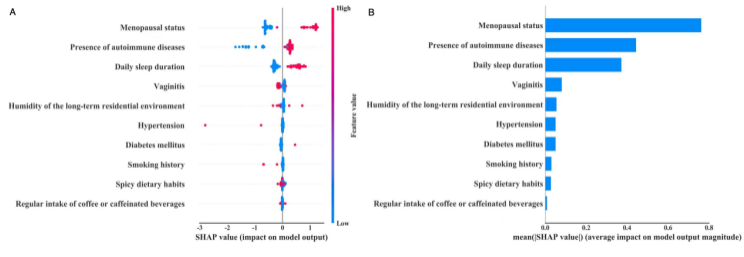
(A) Shapley additive explanations (SHAP) summary plot of relevant factors affecting lichenoid vulvar disease based on the gradient boosting machine model. (B) Importance ranking of relevant factors affecting lichenoid vulvar disease.

To further analyze the impact of multiple factors on the outcome variable within the GBM model, SHAP plots were generated. These plots were constructed to interpret the correlations between various factors and the target variables. Notably, it was observed that menopausal status exhibited the strongest positive dependence with the target variables. Following this, the presence of autoimmune diseases also displayed a positive relevance. Daily sleep duration was positively correlated with LVD, while vaginitis showed a negative association. Further details of the correlation results are presented in Figure 4.

### External Validation of the LVD Warning Model

**Figure 5:**
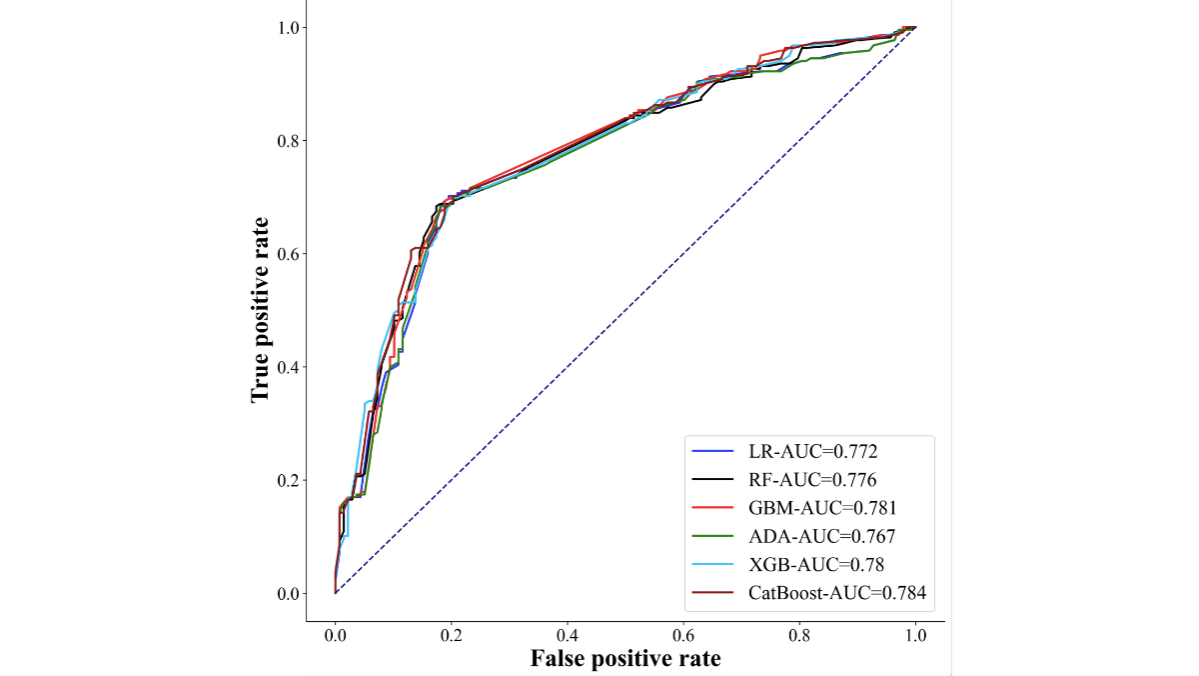
Receiver operating characteristic curves for 6 lichenoid vulvar disease prediction models with external validation. LR: Logistic Regression; RF: Random Forest; GBM: Gradient Boosting Machine; ADA: AdaBoost; XGB: Extreme Gradient Boosting; CatBoost: Categorical Boosting; AUC: Area Under the Curve.

**Figure 6:**
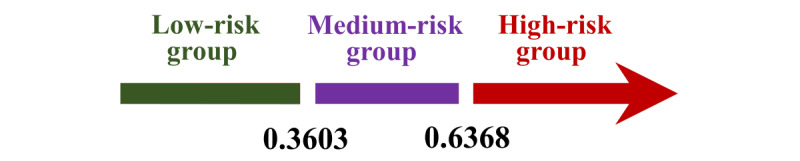
The predictive outcome range of the gradient boosting machine warning model.

**Figure 7:**
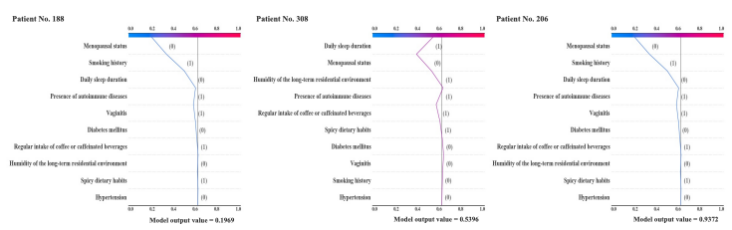
(A) Prediction of low-risk group. (B) Prediction of medium-risk group. (C) Prediction of high-risk group.

To verify the effectiveness of the model, it was first internally validated with the ROC curve, and the results indicated the AUC value of 0.814, as illustrated in Table 2. Furthermore, external validation was performed using baseline data from 356 patients in 2018, and the results showed that the corresponding GBM model achieved an AUC score of 0.778, an accuracy of 0.732, sensitivity of 0.713, and specificity of 0.767. In comparison with the internal validation, the AUC, accuracy, and specificity values only decreased by 0.036, 0.015, and 0.051, respectively, while the sensitivity value improved by 0.1. Therefore, it is evident that the constructed risk warning model exhibits favorable predictive efficacy, as depicted in Figure 5 and Table 3. Afterwards, the GBM warning model was applied to predict the training dataset and obtain predictive probabilities. Subsequently, k-means algorithm was used to cluster those probabilities, yielding 3 cluster centers, 0.45529467, 0.81832383, and 0.26538643. The thresholds for 3 risk zones were calculated as 0.3603 and 0.6368. In short, Figure 6 displays the GBM warning model’s classification of individuals into 3 groups based on predictions, those below 0.3603 as the low-risk group, those above 0.6368 as the high-risk group, and those within the range of 0.3603 to 0.6368 as the medium-risk group. The use of population-related variables as input for the alert model led to the generation of predicted values, depicting varying degrees of risk, as demonstrated in Figure 7.

**Table 3:** Results of the prediction efficacy assessment of 6 lichenoid vulvar disease prediction models.

	Model	Area under the curve, mean (95% CI)	Accuracy, mean (95% CI)	Sensitivity, mean (95% CI)	Specificity, mean (95% CI)	*F*_1_-score, mean (95% CI)
LR^a^		0.778 (0.72-0.818)	0.742 (0.701-0.784)	0.703 (0.645-0.767)	0.808 (0.734-0.864)	0.771 (0.729-0.809)
RF^b^		0.776 (0.735-0.822)	0.722 (0.685-0.764)	0.743 (0.674-0.808)	0.682 (0.628-0.771)	0.765 (0.72-0.808)
GBM^c^		0.778 (0.727-0.822)	0.732 (0.684-0.77)	0.713 (0.667-0.765)	0.767 (0.678-0.826)	0.764 (0.719-0.803)
ADA^d^		0.766 (0.715-0.816)	0.73 (0.682-0.772)	0.713 (0.651-0.772)	0.755 (0.681-0.831)	0.767 (0.714-0.804)
XGB^e^		0.772 (0.722-0.819)	0.712 (0.664-0.763)	0.65 (0.59-0.722)	0.808 (0.744-0.879)	0.734 (0.688-0.784)
CatBoost^f^		0.787 (0.741-0.829)	0.737 (0.681-0.783)	0.696 (0.62-0.754)	0.802 (0.731-0.869)	0.764 (0.708-0.809)

^a^LR: logistic regression.

^b^RF: random forest.

^c^GBM: gradient boosting machine.

^d^ADA: adaptive boosting.

^e^XGB: eXtreme gradient boosting.

^f^CatBoost: categorical boosting.

## Discussion

### Principal Findings

LVD is a common chronic gynecological condition, clinically characterized by vulvar hypopigmentation and pruritus. In middle and later stage, it may lead to dysuria, dyspareunia, introital stenosis, and vulvovaginal atrophy, causing considerable physical and psychological anguish to affected women [18,19]. Many patients experience delayed diagnosis and treatment due to the sensitive nature of the condition, which can cause embarrassment and feelings of shame, or due to misdiagnosis resulting from symptomatic variations. Early misdiagnosis of LVD, particularly VLS, can result in delayed treatment, leading to irreversible vulvar structural abnormalities, scarring, and, in severe cases, genital cancer [20]. Currently, there is no gold-standard treatment for LVD. Conventional first-line therapies are associated with adverse reactions and frequent disease recurrence. Emerging approaches, such as energy-based therapies and platelet-rich plasma injections, have yet to be fully integrated into clinical practice. Therefore, it is of paramount importance to identify factors potentially contributing to LVD in women and establish a predictive model to warn about its risk. To our knowledge, this study is the first systematic investigation of risk factors associated with LVD in women and the development of a predictive model rooted in the principles of evidence-based medicine. Univariate analysis revealed that vaginitis, urinary incontinence, humidity of the long-term residential environment, spicy dietary habits, regular intake of coffee or caffeinated beverages, daily sleep duration, diabetes mellitus, smoking history, presence of autoimmune diseases, menopausal status, and hypertension were all significant risk factors affecting female LVD. Furthermore, the GBM warning model outperforms the other 5 predictive models, demonstrating superior performance with higher ROC curve area, accuracy, sensitivity, and *F*_1_-score. The GBM model reveals that menopausal status has the strongest positive correlation with female LVD, followed by the presence of autoimmune diseases, which also displayed a positive dependency.

### Comparison With Previous Work

The GBM algorithm, a type of boosting algorithm, is capable of exploring the relationships between observed outcomes and various target variables, making it useful for clinical analysis of high-risk factors contributing to inducing the disease [21,22]. In this study, the GBM model was used to construct SHAP plots for analyzing the correlations between multiple factors and the target variables. The results revealed that menopausal status exerted the strongest positive correlation with the development of LVD in women, exhibiting a positive dependency. This finding aligns with the clinical observation that perimenopausal women have a higher incidence of LVD [23]. Günthert et al [24] have reported that the disease may be attributed to endocrine factors, the dearth of sex hormones, as the sex hormone levels in serum of affected individuals are lower than those of normal women of the same age, suggesting that sex hormone may be one of the contributing factors to the pathogenesis of LVD. Furthermore, the presence of autoimmune systemic diseases, daily sleep duration, diabetes, and humidity of the long-term residential environment are all positively correlated with LVD. The skin is a peripheral lymphoid organ with immunological functions that is closely connected to the systemic immune system. A substantial body of evidence has established a close association between LVD and autoimmune disorders. This relationship is particularly evident in cases of vulvar lichen sclerosus, a lymphocyte-mediated chronic localized skin disease, where both cellular and humoral immunity are involved in the pathogenesis. Therefore, patients with LVD often present with various autoimmune diseases, such as vitiligo and hypothyroidism, among others [11,25]. Furthermore, some researches have indicated that diabetes is a systemic metabolic disorder often associated with various skin lesions [26,27]. In particular, individuals with type 2 diabetes are more likely to develop a range of skin disorders. In addition, diabetes tends to exacerbate the pruritus severity in patients with LVD. The intensity of itching is positively correlated with blood glucose levels, indicating that higher blood glucose values result in more pronounced itching symptoms. Similarly, excessive daily sleep duration has been linked to an increased risk of developing type 2 diabetes, thereby further raising the risk of LVD [28,29]. On the other hand, vaginitis showed a negative association with LVD. This could be attributed to the fact that women with vaginitis typically receive prompt and effective diagnosis and treatment, which reduces the risk of overusing medications, for some cases of LVD are due to the overuse of drugs mainly containing aromatics.

### Strengths and Limitations

To sum up, it is crucial for women to cultivate healthy lifestyles and dietary habits, as well as to be informed about the factors related to LVD in order to reduce the incidence of this condition. Furthermore, the study demonstrates that the warning model exhibits strong predictive capabilities and discriminatory effect. The characteristics and strengths of the risk warning model primarily manifest in the following aspects: (1) it is convenient and easy to implement in clinical practice and (2) it can promptly identify the high-risk individuals and provide preventive interventions. To enhance the model’s clinical use, we devised a web-based calculator and mobile app featuring easily accessible functionalities for patients. As a self-diagnostic tool, it offers early detection and timely intervention for LVD.

For female patients identified as low risk for predicted outcomes, it is advisable to undergo appropriate treatment for autoimmune conditions such as thyroid disorders, systemic lupus erythematosus, psoriasis, rheumatism, or arthritis. In addition, managing blood sugar levels, incorporating moderate physical activity, and ensuring adequate sleep duration are recommended. Furthermore, avoiding tight fitting garments, using mild soaps during bathing (avoiding its application on the vulva and instead cleansing the area with water only), gently patting the vulva dry post bathing, and using 100% cotton, unscented or fragrance-free menstrual pads are suggested. Emphasizing guidance on vulvovaginal medications, particularly avoiding products containing fragrances and preservatives, alongside health education, is paramount. Elevating awareness and self-management skills regarding LVD, especially recognizing the risk of neoplastic change associated with the absence of standardized treatment and follow-up, is crucial [7]. Low-risk individuals should maintain annual follow-up appointments.

For individuals categorized as medium-risk, extending lifestyle interventions akin to those recommended for low-risk individuals is advisable. Furthermore, incorporating general management strategies for perimenopause and hormone replacement therapy. In addition, applying preservative-free, unscented or fragrance-free emollient to hold moisture in the skin and improve barrier function is recommended [10]. Medium-risk individuals should maintain biannual follow-up appointments.

For high-risk patients, a higher frequency of follow-ups is recommended, ideally every 3 months. Depending on the situation, timely referral to a higher-level hospital should be considered to implement more precise and personalized treatment and monitoring. In cases where vulvar symptoms are significant, such as persistent itching and lack of self-relief, prompt medical attention is advised. Routine secretion tests should be conducted to rule out herpes simplex and candidal infections, with additional tests for autoimmune diseases, thyroid disease, and diabetes performed as necessary. Dermatoscopy can be used when feasible. After excluding conditions such as vaginitis, vulvar pathological examination may be warranted. In addition, it is advised to avoid any genital skin irritants and allergens, such as cleansing products, frequent water exposure, incontinence, and tight-fitting clothing. Some activities, such as cycling or horse riding, may exacerbate symptoms. Regular application of a barrier emollient to affected areas can protect against local irritants such as urine and menstrual blood [20]. During intercourse, it is recommended to use adequate lubrication, with unscented or fragrance-free silicone-based lubricants. Patients should be advised to closely monitor their vulvar condition and contact their doctor if they notice any changes in appearance or texture, such as lumps, ulceration, or skin hardening, or if there are significant changes in symptoms [10].

### Limitations

This study has a few limitations. It is a single-center cohort study based on data from a single ethnic group, which may limit the generalizability of the findings. In addition, the relatively small sample size could affect the accuracy of the model’s predictions. Future research should aim to include larger sample sizes and to optimize and validate the LVD warning model through multi-center studies involving diverse ethnic and racial groups.

### Conclusions

In conclusion, developing an early warning model for female LVD on the basis of evidence-based medicine, promptly identifying high-risk factors for the condition, and implementing risk-stratified population management are crucial measures to enhance the effectiveness of preventing and treating female LVD. By using the warning model to classify individuals into high-risk and low-risk groups, it is conductive to explore the tailored health intervention strategies, and afford personalized health management recommendations, encompassing adjustments to individual lifestyle habits and the implementation of improved conservative treatment protocols. This contributes to an effect evaluation of grading management mode, facilitating the realization of personalized and long-term pelvic health control patterns. Early detection and prompt treatment of LVD can thus be achieved, thereby ameliorating the quality of life and overall well-being for women.

## Appendix

Multimedia Appendix 1 Characteristics of the included machine learning algorithms and the rationale for their selection.

## Abbreviations

ADA: adaptive boosting
AUC: area under the curve
CatBoost: categorical boosting
GBM: gradient boosting machine
LR: logistic regression
LVD: lichenoid vulvar disease
RF: random forest
ROC: receiver operating characteristic
SHAP: SHapley Additive exPlanations
VLS: vulvar lichen sclerosus
XGB: eXtreme gradient boosting

## Data Availability

Data are available upon request by the corresponding author.

## References

[1] Fruchter R, Melnick L, Pomeranz MK (2017). Lichenoid vulvar disease: a review. Int J Womens Dermatol.

[2] Lee A, Fischer G (2018). Diagnosis and treatment of vulvar lichen sclerosus: an update for dermatologists. Am J Clin Dermatol.

[3] Corazza M, Schettini N, Zedde P, Borghi A (2021). Vulvar lichen sclerosus from pathophysiology to therapeutic approaches: evidence and prospects. Biomedicines.

[4] Ling M, Dongmei W, Yueyue C, Yueting Z, Tao C, Jian M, Xiaoli Z, Yuqing L, Lisha D, Qian W, Tao W, Xiaoyu N (2023). Vaginal microbiota changes in the vulvar lichen simplex chronicus. Clin Exp Obstet Gynecol.

[5] Leis M, Singh A, Li C, Ahluwalia R, Fleming P, Lynde CW (2022). Risk of vulvar squamous cell carcinoma in lichen sclerosus and lichen planus: a systematic review. J Obstet Gynaecol Can.

[6] Steben M (2022). Lichen sclerosus: why do most women struggle with their diagnosis?. J Obstet Gynaecol Can.

[7] American College of Obstetricians Gynecologists' Committee on Practice Bulletins—Gynecology (2020). Diagnosis and management of vulvar skin disorders: ACOG practice bulletin, number 224. Obstet Gynecol.

[8] Sideri M, Parazzini F, Rognoni MT, La Vecchia C, Negri E, Garsia S, Arnoletti E, Cecchetti G (1989). Risk factors for vulvar lichen sclerosus. Am J Obstet Gynecol.

[9] Smith YR, Haefner HK (2004). Vulvar lichen sclerosus : pathophysiology and treatment. Am J Clin Dermatol.

[10] van der Meijden WI, Boffa MJ, Ter Harmsel B, Kirtschig G, Lewis F, Moyal-Barracco M, Tiplica G, Sherrard J (2022). 2021 European guideline for the management of vulval conditions. J Eur Acad Dermatol Venereol.

[11] Tran DA, Tan X, Macri CJ, Goldstein AT, Fu SW (2019). Lichen sclerosus: an autoimmunopathogenic and genomic enigma with emerging genetic and immune targets. Int J Biol Sci.

[12] Virgili A, Borghi A, Cazzaniga S, Di Landro A, Naldi L, Minghetti S, Verrone A, Stroppiana E, Caproni M, Nasca MR, D'Antuono A, Papini M, Di Lernia V, Corazza M, GLS Italian Study Group (2017). New insights into potential risk factors and associations in genital lichen sclerosus: Data from a multicentre Italian study on 729 consecutive cases. J Eur Acad Dermatol Venereol.

[13] Fergus KB, Lee AW, Baradaran N, Cohen AJ, Stohr BA, Erickson BA, Mmonu NA, Breyer BN (2020). Pathophysiology, clinical manifestations, and treatment of lichen sclerosus: a systematic review. Urology.

[14] Gulin SJ, Lundin F, Seifert O (2023). Comorbidity in patients with Lichen sclerosus: a retrospective cohort study. Eur J Med Res.

[15] National Center for Health Statistics National Health Interview Survey.

[16] Damm MMB, Jensen TSR, Mahmood B, Lundh M, Poulsen SS, Bindslev N, Hansen MB (2017). Acetylcholine-related proteins in non-neoplastic appearing colonic mucosa from patients with colorectal neoplasia. Mol Carcinog.

[17] Lee C, Jo B, Woo H, Im Y, Park RW, Park C (2022). Chronic disease prediction using the common data model: development study. JMIR AI.

[18] McCluggage WG (2009). Recent developments in vulvovaginal pathology. Histopathology.

[19] Baggish MS (2016). Fractional CO2 laser treatment for vaginal atrophy and vulvar lichen sclerosus. Journal of Gynecologic Surgery.

[20] Kirtschig G, Kinberger M, Kreuter A, Simpson R, Günthert A, van Hees C, Becker K, Ramakers MJ, Corazza M, Müller S, von Seitzberg S, Boffa MJ, Stein R, Barbagli G, Chi CC, Dauendorffer JN, Fischer B, Gaskins M, Hiltunen-Back E, Höfinger A, Köllmann NH, Kühn H, Larsen HK, Lazzeri M, Mendling W, Nikkels AF, Promm M, Rall KK, Regauer S, Sárdy M, Sepp N, Thune T, Tsiogka A, Vassileva S, Voswinkel L, Wölber L, Werner RN (2024). EuroGuiderm guideline on lichen sclerosus-treatment of lichen sclerosus. J Eur Acad Dermatol Venereol.

[21] Xie Y, Jiang B, Gong E, Li Y, Zhu G, Michel P, Wintermark M, Zaharchuk G (2019). JOURNAL CLUB: use of gradient boosting machine learning to predict patient outcome in acute ischemic stroke on the basis of imaging, demographic, and clinical information. AJR Am J Roentgenol.

[22] Zhang X, Xue Y, Su X, Chen S, Liu K, Chen W, Liu M, Hu Y (2022). A transfer learning approach to correct the temporal performance drift of clinical prediction models: retrospective cohort study. JMIR Med Inform.

[23] Nair PA (2017). Vulvar lichen sclerosus et atrophicus. J Midlife Health.

[24] Günthert AR, Faber M, Knappe G, Hellriegel S, Emons G (2008). Early onset vulvar lichen sclerosus in premenopausal women and oral contraceptives. Eur J Obstet Gynecol Reprod Biol.

[25] Sherman V, McPherson T, Baldo M, Salim A, Gao XH, Wojnarowska F (2010). The high rate of familial lichen sclerosus suggests a genetic contribution: an observational cohort study. J Eur Acad Dermatol Venereol.

[26] Makrantonaki E, Jiang D, Hossini AM, Nikolakis G, Wlaschek M, Scharffetter-Kochanek K, Zouboulis CC (2016). Diabetes mellitus and the skin. Rev Endocr Metab Disord.

[27] de Macedo GMC, Nunes S, Barreto T (2016). Skin disorders in diabetes mellitus: an epidemiology and physiopathology review. Diabetol Metab Syndr.

[28] Zizi F, Jean-Louis G, Brown CD, Ogedegbe G, Boutin-Foster C, McFarlane SI (2010). Sleep duration and the risk of diabetes mellitus: epidemiologic evidence and pathophysiologic insights. Curr Diab Rep.

[29] Briançon-Marjollet A, Weiszenstein M, Henri M, Thomas A, Godin-Ribuot D, Polak J (2015). The impact of sleep disorders on glucose metabolism: endocrine and molecular mechanisms. Diabetol Metab Syndr.

