# Supplementary material for: Development and Validation of a Machine Learning–Based Early Warning Model for Lichenoid Vulvar Disease: Prediction Model Development Study
Source: J Med Internet Res. 2024 Nov 22;26:e55734. doi: 10.2196/55734 (PMC11624445; doi:10.2196/55734)
Supplement: Multimedia Appendix 1 [file jmir_v26i1e55734_app1.docx]

**Characteristics of the included machine learning algorithms and the rationale for their selection:**

Logistic Regression (LR) serves as a fundamental linear classifier, often employed as a baseline model for comparison. In this study, the LR model was implemented using the sklearn 0.22 package in Python 3.7. Parameters were selected based on five-fold cross validation, with max_iter=100 and solver=liblinear chosen.

Random Forest (RF) is a non-linear classifier that aggregates multiple weak classifiers, typically decision trees, through voting to form a robust classifier. It mitigates overfitting by constructing each decision tree based on different random samples and feature subsets, representing the bagging algorithm in ensemble learning. The RF model, implemented using the sklearn 0.22 package in Python 3.7, had parameters selected through five-fold cross validation: n_estimators=50, max_depth=7, min_samples_leaf=4, and min_samples_split=10.

Adaptive Boosting (ADA) is a non-linear classifier that constructs a strong classifier by combining multiple weak classifiers, typically decision trees. It adapts by adjusting the weights of each sample, giving more attention to difficult-to-classify samples in subsequent training. It represents the boosting algorithm in ensemble learning. The ADA model, implemented using the sklearn 0.22 package in Python 3.7, had parameters selected through five-fold cross validation: learning_rate=0.5 and n_estimators=50.

Gradient Boosting Machine (GBM) is a non-linear classifier that that iteratively constructs multiple weak learners, typically decision trees. Each weak learner is trained to minimize the negative gradient of the loss function of the combined model, leading to a reduction in the overall model loss along the direction of the negative gradient upon incorporating the weak learner. It exhibits excellent predictive performance in classification and regression problems, representing the boosting algorithm in ensemble learning. The GBM model, implemented using the sklearn 0.22 package in Python 3.7, had parameters selected through five-fold cross validation: learning_rate=0.1, n_estimators=30, max_depth=4, min_samples_leaf=4, and min_samples_split=10.

eXtreme Gradient Boosting (XGBoost) is a non-linear classifier, akin to GBM, that leverages gradient boosting methodology for model construction. It enhances performance and speed through various techniques like regularization, parallelization, and various tree pruning strategies. Theoretically, XGBoost exhibits superior generalization capability and robustness compared to GBM. The XGBoost model, implemented using the xgboost 1.6.2 package in Python 3.7, had parameters selected through five-fold cross validation: learning_rate=0.1, n_estimators=50, max_depth=6, scale_pos_weight=0.667, and colsample_btree=0.7.

Categorical Boosting (CatBoost) is a nonlinear classifier renowned for its adeptness in efficiently managing categorical features within the gradient boosting framework. It employs symmetric decision trees as sub-trees, ensuring uniformity in the generation of left and right nodes at each layer of splitting (split features and nodes). This strategy aids in mitigating overfitting to some extent while enhancing prediction speed. It represents one of the most advanced machine learning algorithms. The CatBoost model, implemented using the catboost 1.2.5 package in Python 3.7, had parameters selected through five-fold cross-validation: learning_rate=0.1, n_estimators=100, depth=5, and min_data_in_leaf=1.

In summary, the rationale for selecting these models lies in their exemplification of fundamental and archetypal algorithmic types within machine learning: LR for classification model, RF for bagging model, ADA for boosting model, and GBM for gradient boosting tree model. Furthermore, XGBoost and CatBoost represent advanced models that refine and enhance these classic algorithms, showcasing notable prowess, especially within the gradient boosting tree category.
